# Lentivirus-mediated RNAi knockdown of the gap junction protein, Cx43, attenuates the development of vascular restenosis following balloon injury

**DOI:** 10.3892/ijmm.2015.2078

**Published:** 2015-01-23

**Authors:** XIAO-JIAN HAN, MIN CHEN, TAO HONG, LING-YU ZHU, DAN HE, JIU-GENG FENG, LI-PING JIANG

**Affiliations:** 1 Department of Pharmacology, School of Pharmaceutical Science, Nanchang University, Nanchang, Jiangxi 330006, P.R. China; 2 Department of Neurosurgery, The First Affiliated Hospital, Nanchang University, Nanchang, Jiangxi 330006, P.R. China; 3 Institute of Translational Medicine, Nanchang University, Nanchang, Jiangxi 330006, P.R. China; 4 Department of Pharmacy, Zhongshan Hospital of Hubei, Wuhan, Hubei 430033, P.R. China

**Keywords:** gap junction, connexin 43, balloon injury, vascular restenosis, RNA interference

## Abstract

Percutaneous coronary intervention [PCI or percutaneous transluminal coronary angioplasty (PTCA)] has been developed into a mature interventional treatment for atherosclerotic cardiovascular disease. However, the long-term therapeutic effect is compromised by the high incidence of vascular restenosis following angioplasty, and the underlying mechanisms of vascular restenosis have not yet been fully elucidated. In the present study, we investigated the role of the gap junction (GJ) protein, connexin 43 (Cx43), in the development of vascular restenosis. To establish vascular restenosis, rat carotid arteries were subjected to balloon angioplasty injury. At 0, 7, 14 and 2 days following balloon injury, the arteries were removed, and the intimal/medial area of the vessels was measured to evaluate the degree of restenosis. We found that the intimal area gradually increased following balloon injury. Intimal hyperplasia and restenosis were particularly evident at 14 and 28 days after injury. In addition, the mRNA and protein expression of Cx43 was temporarily decreased at 7 days, and subsequently increased at 14 and 28 days following balloon injury, as shown by RT-PCR and western blot analysis. To determine the involvement of Cx43 in vascular restenosis, the lentivirus vector expressing shRNA targeting Cx43, Cx43-RNAi-LV, was used to silence Cx43 in the rat carotid arteries. The knockdown of Cx43 effectively attenuated the development of intimal hyperplasia and vascular restenosis following balloon injury. Thus, our data indicate the vital role of the GJ protein, Cx43, in the development of vascular restenosis, and provide new insight into the pathogenesis of vascular reste-nosis. Cx43 may prove to be a novel potential pharmacological target for the prevention of vascular restenosis following PCI.

## Introduction

Coronary artery disease (CAD) is one of the most common cardiovascular diseases in humans with a high incidence of morbidity and mortality. It is well recognized that coronary artery atherosclerosis is the vital cause of CAD, and CAD has become a major public health issue worldwide. Since its first introduction in 1977, percutaneous coronary intervention [PCI or percutaneous transluminal coronary angioplasty (PTCA)] has been developed into one of the main therapeutic strategies for CAD (1). PCI improves coronary blood flow, reduces angina pectoris and improves the quality of life of patients (2). Moreover, PCI is relatively easy to perform and causes minimal injury. However, the high incidence of restenosis (RS) following PCI usually limits its clinical effect. The incidence of RS is approximately 20–50% at 6 months following PCI and with the implantation of the stent, the incidence of RS is approximately 25–30% (3). Thus, vascular RS is a serious complication of PCI, and thus the development of novel therapeutic strategies for the prevention of RS is urgently required.

It has been well documented that the proliferation and migration of vascular smooth muscle cells (VSMCs) is a key factor in the development of RS (4). VSMCs in mature animals are highly differentiated, and their principal function is contraction. However, VSMCs can also proliferate and produce the matrix components of the blood vessel wall during vasculogenesis (4). In addition, VSMCs retain remarkable plasticity, and can undergo relatively rapid and reversible changes in their phenotype in response to local environmental stress (5). Vascular RS at an early stage can be induced by vasospasm or decreased vessel elasticity several hours or several days following PCI. Subsequently, the damaged endothelial cells and macrophages invade the endothelial sublayer and release cytokines, including endothelin, angiotensin II, basic fibroblast growth factor, platelet-derived growth factor and transforming growth factor. These cytokines stimulate the migration and proliferation of VSMCs and induce the accumulation of extracellular matrix components, which results in the remodeling of the blood vessel wall and vascular RS (6). Although some intracellular signaling pathways associated with the proliferation and migration of VSMCs have been identified (2,7,8), the role of direct intercellular communication pathways, gap junctions (GJs), in the development of RS requires further investigation.

GJs are plasma membrane spatial microdomains constructed of assemblies of channel proteins termed connexins in vertebrates and innexins in invertebrates (9). The channels provide direct intercellular communication pathways allowing the rapid exchange of ions, second messengers and small metabolites of up to 1 kDa in molecular mass (10). *In vitro* studies have demonstrated that the permeability, conductance and other properties of GJ channels depend on the precise make-up of their component connexins (11). In the major arteries, endothelial GJs may simultaneously express 3 connexin isotypes, connexin (Cx)40, Cx37 and Cx43, whereas VSMCs predominantly express Cx43 and, in some instances, Cx40 or Cx45 (12–14). It has been found that Cx43 expression is significantly increased during the alteration of the VSMC phenotype (15). Furthermore, the size, quantity, distribution and structure of Cx43 in vascular lesions may also be altered, which is known as Cx43 remodeling (16). It has been demonstrated that Cx43 remodeling affects not only the conductivity and permeability of the GJ itself, but also the electrical, chemical and metabolic channels between adjacent cells (17–19). On the other hand, Cx43 remodeling has also been shown to play a crucial role in the pathogenesis of cardiovascular diseases (20).

In the present study, we established a model of vascular RS by subjecting rat carotid arteries to angioplasty balloon injury to mimic the development of RS following PCI. The results revealed that the intimal area of the arteries gradually increased following balloon injury. Simultaneously, the mRNA and protein expression of Cx43 was also increased during the development of RS. Importantly, the knockdown of Cx43 effectively prevented the development of intimal hyperplasia and vascular RS following balloon injury. Thus, our data indicate the vital role of the GJ protein, Cx43, in the development of vascular RS, and may thus provide a novel potential pharmacological target for the prevention of vascular RS following PCI.

## Materials and methods

### Experimental animals

Male Sprague-Dawley rats (purchased from the Department of Animal, Nanchang University, Nanchang, China) weighing 300–400 g were maintained on a regular chow diet prior to the study. All procedures for the animal experiments were carried out in accordance with the National Institutes of Health Guidelines, and were approved by the Ethics Committee for Animal Axperiments of Nanchang University.

### Establishment of model of vascular RS by balloon injury

The rats were anesthetized with an intraperitoneal injection of Hydral (10%, 3.5 ml/kg; Harbin Pharmaceutical Group Co., Ltd., Harbin, China). To establish the model of vascular RS, the angioplasty balloon (1.5×20 mm; Cordis Corp., Miami, FL, USA) was inserted into the rat common carotid artery through an incision in the left external carotid artery, as previously described (21). The balloon was then sufficiently inflated in the carotid artery and was drawn 3 times consistently from the proximal area to the carotid bifurcation to produce endothelial denudation. The external carotid was ligated and blood flow in the common carotid was restored. In addition, the rats were intramuscularly injected with benzylpenicillin sodium (40×10^4^ IU/day for 3 days; Harbin Pharmaceutical Group Co., Ltd.) to prevent infection. The rats were euthanized by an overdose of Hydral at 0, 7, 14 and 28 days (n=6/group) following balloon injury. The injured common carotid arteries were collected for hematoxylin and eosin (H&E) staining or western blot analysis to evaluate vascular remodeling and the expression of Cx43 following balloon injury.

### H&E staining

Three serial cryosections (at 5-*μ*m-thick) were prepared from the middle portion of the rat injured common carotid arteries. The slices were stained with H&E (Sangon Biotech, Shanghai, China) and histomorphological observation was performed under a light microscope (Olympus, Tokyo, Japan) to examine the structure of the blood vessel wall following balloon injury. The intimal and medial area of the 3 serial cryosections in each sample were measured using Image-Pro Plus 5.0 software (Media Cybernetics, Inc., Houston, TX, USA).

### Reverse transcription-polymerase chain reaction (RT-PCR)

Total RNA was extracted from the rat carotid tissue using TRIzol reagent (Tiangen, Beijing, China) and cDNA was synthesized from the extracted total RNA using the SuperScript III kit (Promega, Madison, WI, USA) following the manufacturer’s instructions. The specific primers used for PCR were as follows: Cx43 sense, 5′-AAAGGCGTTAAGG ATCGCGTG-3′ and antisense, 5′-GTCATCAGGCCGAGG CCT-3′ [as previously described (23)]; β-actin sense, 5′-CCCA TCTATGAGGGTTACGC-3′ and antisense, 5′-TTTAATGT CACGCACGATTTC-3′. All specific primers were chemically synthesized (Generay Biotech Co. Ltd., Shanghai, China). The PCR reactions were performed using the GeneAmp PCR System 9700 (Applied Biosystems, Foster City, CA, USA). The amplification conditions for Cx43 was as follows: 94°C for 2 min followed by 32 cycles at 94°C for 45 sec, 58°C for 45 sec, and 72°C for 90 sec, and a final extension at 72°C for 5 min. The PCR conditions for β-actin was as follows: 94°C for 2 min followed by 28 cycles at 94°C for 30 sec, 56°C for 30 sec, and 72°C for 90 sec, and a final extension at 72°C for 5 min. The amplified RT-PCR products were separated on 1.2% (w/v) agarose containing ethidium bromide (both from Sangon Biotech) for 30 min. The results of electro phoresis were photographed was using the Molecular Imager^®^ Chemi DOC^T^XRS^+^ system, and the signal densities of the gels were analyzed using Quantity One software (both from Bio-Rad, Hercules, CA, USA).

### Western blot analysis

The carotid arteries were pulverized in radioimmunoprecipitation assay (RIPA) lysis buffer (Sangon Biotech) using a homogenizer. The whole lysate was centrifuged at 12,000 rpm for 10 min at 4°C, and the pellets were resuspended in sample buffer containing 4% sodium dodecyl sulfate (SDS; Sangon Biotech). Proteins were separated by SDS-polyacrylamide gel electrophoresis (PAGE) and transferred onto nitrocellulose membranes (Millipore, Bedford, MA, USA). The membranes were blocked in 5% skim milk for 1 h at room temperature, and were incubated with rabbit polyclonal anti-Cx43 antibody (zym-71-0700; 1:250 in 5% skim milk; Zymed Laboratories, San Francisco, CA, USA) or rabbit monoclonal anti-glyceraldehyde 3-phosphate dehydrogenase (GAPDH) antibody (MAB374; 1:1,000 in 5% skim milk; Chemicon, Temecula, CA, USA) overnight at 4°C. The membranes were then washed 3 times with TBST, then incubated with the relative HRP-conjugated IgG secondary antibody (ZB-5301 and ZB-2305; Zhongshan Golden Bridge Biotechnology Co., Ltd., Beijing, China) for 2 h at room temperature and washed in TBST 3 times. Chemiluminescence were carried out using Amersham ECL Prime Western Blotting Detection reagents, and the immunobloting signal was detected using the Molecular Imager Chemi DOC^T^XRS^+^ system (Bio-Rad). The intensity of each Cx43 band was normalized to the GAPDH band, and the relative expression of Cx43 following balloon injury was normalized to the control.

### Construction of GFP-Cx43-shRNA-lentiviral vectors

In order to examine the role of Cx43 in the development of vascular RS, the lentivirus expressing shRNA targeting Cx43, Cx43-RNAi-LV, was constructed (GeneChem, Shanghai, China). In brief, the shRNA sequence for Cx43 (5′-AGAGCACGGCAAGGTGAAA-3′) was designed using the manufacturer’s RNA interference (RNAi) Designer program, and the negative control construct (control shRNA) was created using a scrambled sequence (5′-TTCTCCGAACGTGTCACGT-3′), as previously described (22). DNA oligos were chemically synthesized (GeneChem, Shanghai, China), annealed and inserted into the expression vector by double digestion with *Age* I and *Eco* RI (New England Biolabs, Ipswich, MA, USA), and ligated with T4 DNA ligase (Takara, Dalian, China) in accordance to the manufacture’s instructions. The ligation was transformed into competent *E. coli* cells and then confirmed by restriction enzyme analysis and DNA sequencing. The sequences were then cloned into pGCSIL-GFP to generate lentiviral vectors. The expression vectors and package vectors were then transfected into HEK293T cells (ATCC, Manassas, VA, USA) using Lipofectamine 2000 (Invitrogen, Carlsbad, CA, USA). Following 48 h of culture, the supernatants containing the lentiviruses, such as Cx43-RNAi-LV and NC-GFP-LV (negative control) were harvested. Purification was then performed using ultracentrifugation and the lentiviral titer was determined.

### Lentivirus-mediated RNAi knockdown of Cx43

Firstly, the knockdown efficiency of Cx43 by the lentiviral vector, Cx43-RNAi-LV, was confirmed in NRK-52E cells (ATCC). In brief, NRK-52E cells at 30% confluence were infected with Cx43-RNAi-LV and NC-GFP-LV, and the cells were then harvested 7 days following infection to obtain whole cell lysate. Following electrophoresis of the cell lysate, the expression level of Cx43 in each group was examined by western blot analysis. To determine the effect of Cx43-RNAi-LV on the balloon injury-induced expression of Cx43 in the arteries, the rats were randomly divided into 4 groups (n=6 in each group) as follows: i) the control group (no balloon injury); ii) the untreated group with ballon injury (balloon injury only); iii) the group with ballon injury treated with Cx43-RNAi-LV (balloon injury + Cx43-RNAi-LV); and iv) the group with ballon injury treated with NC-GFP-LV (balloon injury + NC-GFP-LV). In order to knockdown Cx43 in the VSMCs, the lentivirus (5×10^8^ TU/ml, 100 *μ*l) expressing shRNA or the scrambled RNA was injected into the balloon-injured rat carotid arteries using a polyethylene catheter. The rats were euthanized by an overdose of Hydral 28 days following balloon injury, and the injured carotid arteries were collected for H&E staining or western blot analysis to evaluate vascular remodeling and the knockdown efficiency of Cx43.

### Statistical analysis

The quantitative data are presented as the means ± standard deviation (SD). Data were analyzed using either the Student’s t-test to compare 2 conditions or ANOVA followed by planned comparisons of multiple conditions. A value of P<0.05 was considered to indicate a statistically significant difference.

## Results

### Confirmation of vascular RS following balloon injury

**Figure 1:**
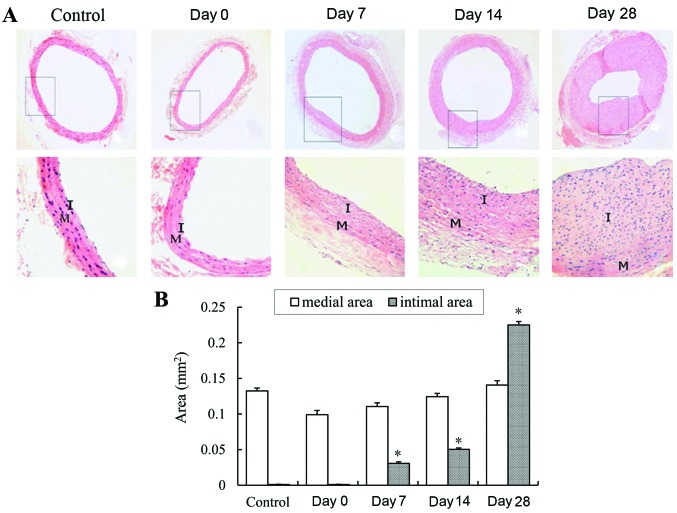
Intimal hyperplasia and restenosis (RS) were induced in rat carotid arteries following balloon injury. (A) Histomorphological observation of control (no injury) and balloon-injured arterial sections. Carotid arteries were removed from the control or balloon-injured rats at the indicated time points. Following hematoxylin and eosin (H&E) staining, the general histological features of the arterial sections at 5 *μ*m were examined under a microscope. Images at low and high magnification are shown in the upper and lower panel, respectively (upper panel, x100 magnification; bottom panel, x400 magnification). I, intima; M, media. (B) The intimal and medial area of the arterial sections was measured using Image-Pro Plus 5.0 software. Data represent the means of 6 independent experiments. ^*^P<0.01 vs. control.

Balloon injury was inflicted to the rat common carotid arteries for the establishment of a model of vascular RS. At 0, 7, 14 and 28 days following balloon injury, the rats were sacrificed by an overdose of Hydral. The carotid arteries were removed and subjected to cryosection at 5 *μ*m. A histomorphological observation of the arterial sections with H&E staining was carried tou to evaluate RS at different time points following balloon injury. Neointima formation occurred in the injured arteries at 7 days (Fig. 1A). Intimal hyperplasia and RS were particularly evident at 14 and 28 days following balloon injury. Compared with the control arteries, the intimal area of the arteries was significantly increased at 14 and 28 days following balloon injury (Fig. 1B). These histomorphological changes in the carotid arteries indicated that the model of vascular RS induced by balloon injury was successful established.

### mRNA and protein expression of Cx43 during the development of RS following balloon injury

**Figure 2:**
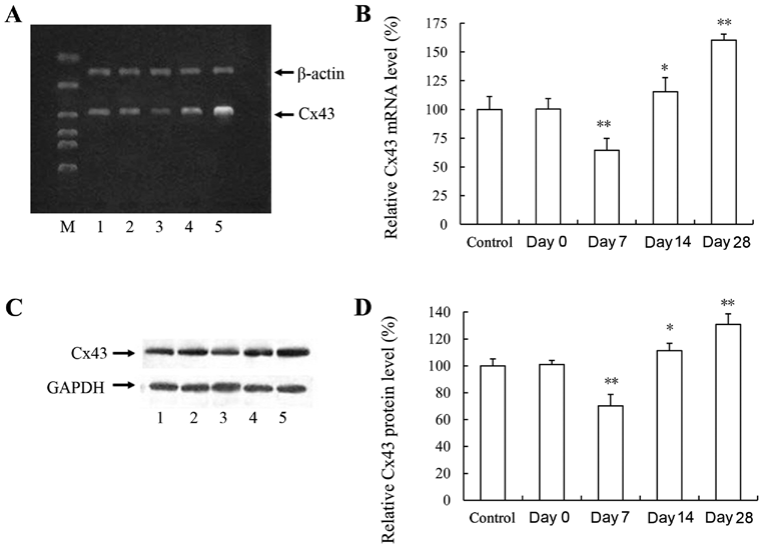
mRNA and protein expression of connexin 43 (Cx43) during the development of restenosis (RS) following balloon injury. (A) Electrophoresis of the RT-PCR product of Cx43 and β-actin on an agarose gel. Lanes 1–5 represent samples from the control, and days 0, 7, 14 and 28, respectively. Lane M represents DNA Marker. β-actin was used as an endogenous reference. DNA ladder was 2,000, 1,000, 750, 500, 250 and 100 bp from top to bottom, respectively. (B) Quantification of relative Cx43 mRNA level was indicated as the normalization of ratio of Cx43/β-actin in each sample relative to the control. Data represent the means of at least 3 independent experiments. ^*^P<0.05 and ^**^P<0.01 vs. control. (C) Western blot analysis of Cx43 protein expression during the development of RS following balloon injury. Lanes 1–5 represent samples from the control, and days 0, 7, 14 and 28, respectively. GAPDH was used as an endogenous reference. (D) Quantification of relative Cx43 expression was indicated as the normalization of ratio of Cx43/GAPDH in each sample to relative to the control. Data represent the means of at least 3 independent experiments. ^*^P<0.05 and ^**^P<0.01 vs. control.

To investigate the role of the GJ protein, Cx43, in the development of vascular RS, the mRNA and protein expression of Cx43 in the arteries following balloon injury was examined by RT-PCR and western blot analysis, respectively. Compared with the controls, the mRNA expression of Cx43 was temporarily decreased at 7 days, and was subsequently increased at 14 and 28 days following balloon injury (Fig. 2A and B). Similarly, the protein expression of Cx43 was significantly upregulated at 14 and 28 days following balloon injury, although a transient decrease in Cx43 expression was detected at 7 days (Fig. 2C and D). These results suggest that the GJ protein, Cx43, is involved in the development of intimal hyperplasia and vascular RS following balloon injury.

### Lentivirus Cx43-RNAi-LV for the specific knockdown of Cx43

**Figure 3:**
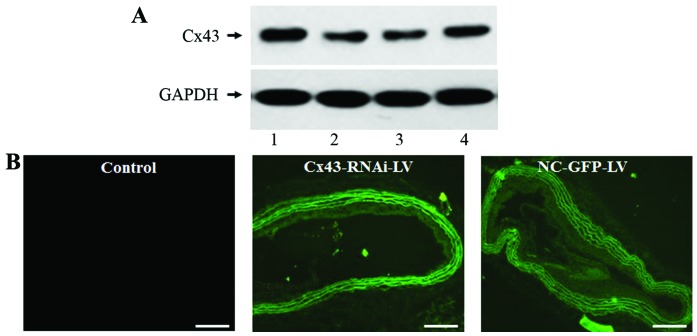
Lentivirus-mediated RNAi knockdown of connexin 43 (Cx43) *in vitro* and the infection efficiency of the lentivirus vector in injured arteries. (A) Knockdown of Cx43 in NRK-52E cells by Cx43-RNAi-LV. Following infection with the lentiviral vectors, Cx43-RNAi-LV or NC-GFP-LV, the cells were harvested and the expression of Cx43 in each group was examined by western blot analysis. GAPDH was used as an endogenous reference. Lanes 1–4 represent samples from the control, 2.5×10^8^ TU/ml Cx43-RNAi-LV-treated cells, 5×10^8^ TU/ml Cx43-RNAi-LV-treated cells and NC-GFP-LV-treated cells, respectively. (B) Infection with Cx43-RNAi-LV and NC-GFP-LV in arteries. Following infection with lentivirus, the fluorescence signal of GFP in the arteries was examined under a fluorescence microscope to show the infection of Cx43-RNAi-LV or NC-GFP-LV. Scale bar, 100 *μ*m.

In order to directly demonstrate the role of Cx43 in the development of vascular RS following balloon injury, the lentiviral vector expressing shRNA targeting Cx43 (Cx43-RNAi-LV) and the negative control lentiviral vector (NC-GFP-LV) were constructed. *In vitro* experiments revealed that the expression of Cx43 was specifically decreased in the NRK-52E cells infected with Cx43-RNAi-LV, but not in the NC-GFP-LV-infected cells (Fig. 3A). These results indicated that the lentiviral vector, Cx43-RNAi-LV, was effective in silencing Cx43. For the knockdown of Cx43 *in vivo*, the injured rat arteries were infected with Cx43-RNAi-LV or NC-GFP-LV, and the infection efficiency *in vivo* was examined by measuring the fluorescent signal of GFP. The green fluorescence was strongly observed in the neointima and media in the Cx43-RNAi-LV-treated and NC-GFP-LV-treated arteries, but not in the controls (Fig. 3B). This indicated that the balloon-injured rat carotid arteries were successfully infected with the lentivirus.

### Lentivirus-mediated RNAi knockdown of Cx43 attenuates the development of intimal hyperplasia and vascular RS induced by balloon injury

**Figure 4:**
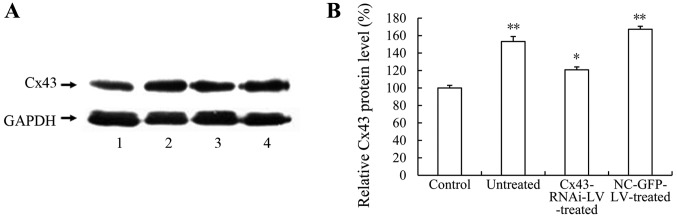
Connexin 43 (Cx43)-RNAi-LV significantly inhibits the balloon injury-induced expression of Cx43 in arteries. (A) Expression of Cx43 in arteries. Lanes 1–4 represent samples from the control, balloon-injured arteries, Cx43-RNAi-LV + balloon injury and NC-GFP-LV + balloon injury, respectively. GAPDH was used as an endogenous reference. (B) Quantification of Cx43 expression was indicated as the normalization of ratio of Cx43/GAPDH in each sample relative to the control. Data represent the means of at least 3 independent experiments. ^**^P<0.01 vs. control (no injury); ^*^P<0.05 vs. untreated group.

**Figure 5:**
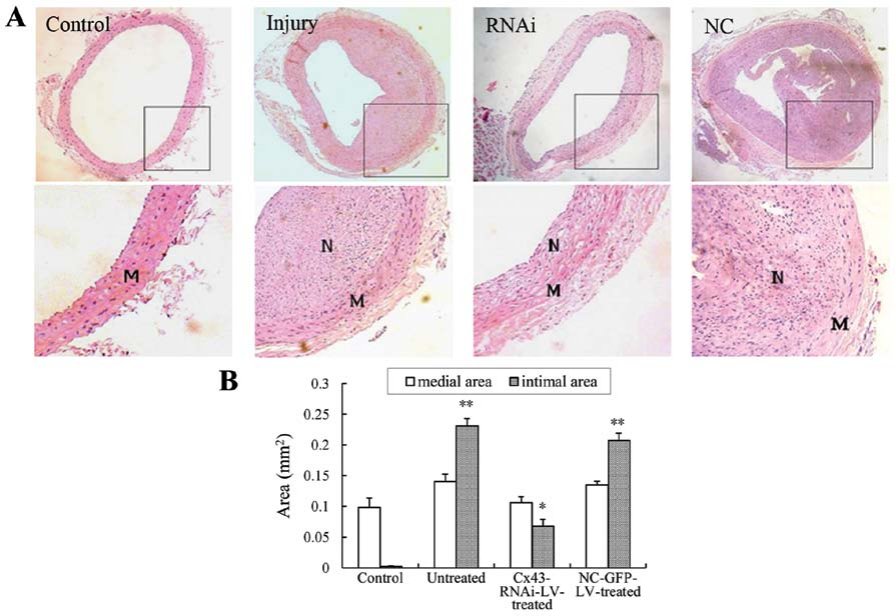
Connexin 43 (Cx43)-RNAi-LV effectively attenuates the development of intimal hyperplasia and restenosis (RS) in arteries following balloon injury. (A) Histomorphological observation of arteries after the indicated treatments. Carotid arteries were removed after the indicated treatments, and were subjected to cryosections at 5 *μ*m. Following hematoxylin and eosin (H&E) staining, the general histological features of the arterial sections were examined under a microscope. Images at low and high magnification are shown in upper and lower panels, respectively (upper panel, x100 magnification; bottom panel, x400 magnification). N, neointima; M, muscle. (B) The intimal and medial area of the arterial sections was measured using Image-Pro Plus 5.0 software. Data represent the means of 6 independent experiments. ^**^P<0.01 vs. control (no injury); ^*^P<0.05 vs. untreated group.

Since the mRNA and protein expression of Cx43 was upregulated during the development of vascular RS (Fig. 2), we firstly examined the effect of Cx43-RNAi-LV on the balloon injury-induced expression of Cx43. Compared with the untreated or NC-GFP-LV-treated arteries, infection with Cx43-RNAi-LV significantly decreased the balloon injury-induced expression of Cx43 (Fig. 4). In addition, the histomorphological observation of the arterial sections (Fig. 5A) and statistical analysis of the intimal/medial areas (Fig. 5B) revealed that the lentivirus-mediated RNAi knockdown of Cx43 significantly attenuated balloon injury-induced intimal hyperplasia and RS. These results directly demonstrate that the GJ protein, Cx43, contributes to the development of vascular RS following balloon injury, although the underlying mechanisms require further investigation.

## Discussion

PCI is one of the main therapeutic strategies used for the treatment of CAD. However, the high incidence of vascular RS limits its clinical effect, and the specific mechanisms responsible for the development of RS following PCI have not yet been fully elucidated. It is acknowledged that vascular RS is mainly caused by endothelial denudation, as well as by the proliferation and migration of VSMCs (1,2). On the other hand, increasing evidence suggests that GJs, a basic structure between cells, may be involved in the proliferation and migration-related signaling pathways of VSMCs (22,24). In previous studies, it was found that alterations in connexin expression correlated with the development of certain vascular diseases, such as hypertension, atherosclerosis and RS (25,26). Notably, the alteration of Cx43 in size, quantity, distribution and structure strikingly influences the conductivity and permeability of GJs, which changes the electrical, chemical and metabolic channels between cells, and subsequently causes ‘selective filtration’ in transmitting information (17–19). In a previous study, in transgenic mice, it was found that a decrease in Cx43 expression effectively inhibited acute neointimal formation in mice with hypercholesterolemia (27). Therefore, the remodeling of Cx43 may play an important role in the pathogenesis of vascular diseases, including hypertension, atherosclerosis and RS. It should be of interest to investigate whether the remodeling of Cx43 is also involved in the development of intimal hyperplasia and vascular RS following PCI.

In the present study, we established a model of vascular RS by subjecting rat common carotid arteries to balloon injury to mimic the development of RS following PCI. Neointimal formation was evident at 7 days following balloon injury (Fig. 1). The stenosis of the vessel lumen was evident, and much more neointimal formation was observed at 14 days following injury. Notably, the lumen area was significantly reduced, and neointimal hyperplasia was also markedly evident at 28 days following balloon injury. These results indicated that balloon injury successfully reproduced RS, and mimicked the development of RS following PCI. On the other hand, we examined the mRNA and protein expression of Cx43 during the development of RS following balloon injury. It was found that the mRNA and protein level of Cx43 was temporarily decreased at 7 days following balloon injury, which may be caused by tissue damage, stress response or a variety of growth factors released from mechanically injured VSMCs at the early stage of injury (6). By contrast, the mRNA and protein expression of Cx43 was significantly upregulated at 14 and 28 days following injury (Fig. 2). These results are consistent with those of previous studies (28,29), and suggest the involvement of Cx43 in the development of intimal hyperplasia and RS following balloon injury. To obtain direct evidence that Cx43 contributes to the development of RS following balloon injury, a lentiviral vector expressing shRNA was used to silence Cx43 in rat carotid arteries (Fig. 3). Compared with the control or NC-GFP-LV group, infection with Cx43-RNAi-LV was effective for the specific knockdown of Cx43 in the NRK-52E cells *in vitro* (Fig. 3A). In addition, it was found that Cx43-RNAi-LV effectively decreased the expression of Cx43 in the injured arteries *in vivo* (Fig. 4). Importantly, the lentivirus-mediated RNAi knockdown of Cx43 effectively inhibited the development of intimal hyperplasia and RS following balloon injury (Fig. 5). Taken together, these results indicate that Cx43 remodeling is involved in the pathogenesis of RS in balloon-injured arteries, and strongly suggests the vital role of Cx43 in the development of RS following PCI. Although the specific mechanisms of action of Cx43 in the development of RS remain unclear, it is hypothesized that the renin-angiotensin-aldosterone system (RAAS) and the mitogen-activated protein kinase (MAPK) signaling pathway mediate the involvement of Cx43 in the development of intimal hyperplasia and RS. It has previously been demonstrated that RAAS is activated following balloon injury and that angiotensin Ⅱ significantly induces neointimal hyperplasia (7). In human saphenous vein smooth muscle cells (SMCs), angiotensin II has also been shown to significantly induce the expression of Cx43 through the angiotensin II type I receptor. Silencing Cx43 inhibits the angiotensin II-induced proliferation and migration of SMCs. In addition, the inhibition of the MAPK-AP-1 signaling pathway efficiently attenuates the angiotensin II-induced Cx43 expression and proliferation of SMCs (8). Therefore, the involvement of Cx43 remodeling in the development of intimal hyperplasia and RS following balloon injury may be mediated by the RAAS or MAPK-AP-1 signaling pathway.

In conclusion, to the very best of our knowledge, our data demonstrate for the first time that the knockdown of the GJ protein, Cx43, may be an effective strategy to prevent the development of intimal hyperplasia and RS following acute injury to rat carotid arteries. In the present study, we reproduced vascular RS by balloon angioplasty injury. The mRNA and protein expression levels of Cx43 were temporarily decreased, and subsequently increased during the development of balloon injury-induced intimal hyperplasia and RS. The knockdown of Cx43 with the lentiviral vector, Cx43-RNAi-LV, significantly inhibited the balloon injury-induced expression of Cx43, and also effectively attenuated the development of intimal hyper-plasia and RS in the arteries following balloon injury. These results indicate that the GJ protein, Cx43, plays an important role in the pathogenesis of RS in injured arteries. Therefore, our data suggest that Cx43 may be a novel and promising pharmacological target for the prevention of intimal hyperplasia and RS following PCI.

## Abbreviations

PCI: percutaneous coronary intervention
RS: restenosis
VSMCs: vascular smooth muscle cells
GJs: gap junctions
Cx43: connexin 43
RNAi: RNA interference
CAD: coronary artery disease
RAAS: renin-angiotensin-aldosterone system

## References

[1] Trikalinos TA, Alsheikh-Ali AA, Tatsioni A, Nallamothu BK, Kent DM (2009). Percutaneous coronary interventions for non-acute coronary artery disease: a quantitative 20-year synopsis and a network meta-analysis. Lancet.

[2] Meads C, Cummins C, Jolly K, Stevens A, Burls A, Hyde C (2000). Coronary artery stents in the treatment of ischaemic heart disease: a rapid and systematic review. Health Technol Assess.

[3] Odell A, Grip L, Hallberg LR (2006). Restenosis after percutaneous coronary intervention (PCI): experiences from the patients’ perspective. Eur J Cardiovasc Nurs.

[4] Zhang C, Chaturvedi D, Jaggar L, Magnuson D, Lee JM, Patel TB (2005). Regulation of vascular smooth muscle cell proliferation and migration by human sprouty 2. Arterioscler Thromb Vasc Biol.

[5] Owens GK (1995). Regulation of differentiation of vascular smooth muscle cells. Physiol Rev.

[6] Crowley ST, Ray CJ, Nawaz D, Majack RA, Horwitz LD (1995). Multiple growth factors are released from mechanically injured vascular smooth muscle cells. Am J Physiol.

[7] Li F, Zhang C, Schaefer S, Estes A, Malik KU (2005). ANG II-induced neointimal growth is mediated via cPLA2- and PLD2-activated Akt in balloon-injured rat carotid artery. Am J Physiol Heart Circ Physiol.

[8] Jia G, Cheng G, Gangahar DM, Agrawal DK (2008). Involvement of connexin 43 in angiotensin II-induced migration and proliferation of saphenous vein smooth muscle cells via the MAPK-AP-1 signaling pathway. J Mol Cell Cardiol.

[9] Hervé JC, Phelan P, Bruzzone R, White TW (2005). Connexins, innexins and pannexins: bridging the communication gap. Biochim Biophys Acta.

[10] Bruzzone R, White TW, Paul DL (1996). Connections with connexins: the molecular basis of direct intercellular signaling. Eur J Biochem.

[11] Elfgang C, Eckert R, Lichtenberg-Fraté H (1995). Specific permeability and selective formation of gap junction channels in connexin -transfected HeLa cells. J Cell Biol.

[12] van Kempen MJ, Jongsma HJ (1999). Distribution of connexin37, connexin40 and connexin43 in the aorta and coronary artery of several mammals. Histochem Cell Biol.

[13] Hong T, Hill CE (1998). Ristricted expression of the gap junctional protein connexin43 in the arterial system of the rat. J Anat.

[14] Li X, Simard JM (2002). Increase in Cx45 gap junction channels in cerebral smooth muscle cells from SHR. Hypertension.

[15] Matsushita T, Rama A, Charolidi N, Dupont E, Severs NJ (2007). Relationship of connexin43 expression to phenotypic modulation in cultured human aortic smooth muscle cells. Eur J Cell Biol.

[16] Ram R, Wescott AP, Varandas K, Dirksen RT, Blaxall BC (2014). Mena associates with Rac1 and modulates connexin 43 remodeling in cardiomyocytes. Am J Physiol Heart Circ Physiol.

[17] Kieken F, Mutsaers N, Dolmatova E (2009). Structural and molecular mechanisms of gap junction remodeling in epicardial border zone myocytes following myocardial infarction. Circ Res.

[18] Qu J, Volpicelli FM, Garcia LI (2009). Gap junction remodeling and spironolactone-dependent reverse remodeling in the hypertrophied heart. Circ Res.

[19] Rucker-Martin C, Milliez P, Tan S (2006). Chronic hemodynamic overload of the atria is an important factor for gap junction remodeling in human and rat hearts. Cardiovasc Res.

[20] Sovari AA, Rutledge CA, Jeong EM (2013). Mitochondria oxidative stress, connexin43 remodeling, and sudden arrhythmic death. Circ Arrhythm Electrophysiol.

[21] Meng QH, Yang G, Yang W, Jiang B, Wu L, Wang R (2007). Protective effect of hydrogen sulfide on balloon injury-induced neointima hyperplasia in rat carotid arteries. Am J Pathol.

[22] Ai Z, Yin L, Zhou X (2006). Inhibition of survivin reduces cell proliferation and induces apoptosis in human endometrial cancer. Cancer.

[23] Barac YD, Zeevi-Levin N, Yaniv G (2005). The 1,4,5-inositol trisphosphate pathway is a key component in Fas-mediated hypertrophy in neonatal rat ventricular myocytes. Cardiovasc Res.

[24] Chadjichristos CE, Morel S, Derouette JP (2008). Targeting connexin 43 prevents platelet-derived growth factor-BB-induced phenotypic change in porcine coronary artery smooth muscle cells. Circ Res.

[25] Severs NJ, Rothery S, Dupont E (2001). Immunocytochemical analysis of connexin expression in the healthy and diseased cardiovascular system. Microsc Res Tech.

[26] Brisset AC, Isakson BE, Kwak BR (2009). Connexins in vascular physiology and pathology. Antioxid Redox Signal.

[27] Chadjichristos CE, Matter CM, Roth I (2006). Reduced connexin43 expression limits neointima formation after balloon distension injury in hypercholesterolemic mice. Circulation.

[28] Wang L, Chen J, Sun Y (2005). Regulation of connexin expression after balloon injury: possible mechanisms for antiproliferative effect of statins. Am J Hypertens.

[29] Wang LH, Chen JZ, Sun YL (2005). Statins reduce connexin40 and connexin43 expression in atherosclerotic aorta of rabbits. Int J Cardiol.

